# Participatory Surveillance Based on Crowdsourcing During the Rio 2016 Olympic Games Using the Guardians of Health Platform: Descriptive Study

**DOI:** 10.2196/16119

**Published:** 2020-04-07

**Authors:** Onicio Leal Neto, Oswaldo Cruz, Jones Albuquerque, Mariana Nacarato de Sousa, Mark Smolinski, Eduarda Ângela Pessoa Cesse, Marlo Libel, Wayner Vieira de Souza

**Affiliations:** 1 University of Zurich Zurich Switzerland; 2 Epitrack Recife Brazil; 3 Scientific Computation Program Oswaldo Cruz Foundation Rio de Janeiro Brazil; 4 Immunopathology Lab Keizo Asami Recife Brazil; 5 Ending Pandemics San Francisco, CA United States; 6 Aggeu Magalhães Research Center Oswaldo Cruz Foundation Recife Brazil

**Keywords:** participatory surveillance, epidemiology, infectious diseases, pandemics, health innovation, digital disease detection, disease surveillance, mobile phone

## Abstract

**Background:**

With the evolution of digital media, areas such as public health are adding new platforms to complement traditional systems of epidemiological surveillance. Participatory surveillance and digital epidemiology have become innovative tools for the construction of epidemiological landscapes with citizens’ participation, improving traditional sources of information. Strategies such as these promote the timely detection of warning signs for outbreaks and epidemics in the region.

**Objective:**

This study aims to describe the participatory surveillance platform Guardians of Health, which was used in a project conducted during the 2016 Olympic and Paralympic Games in Rio de Janeiro, Brazil, and officially used by the Brazilian Ministry of Health for the monitoring of outbreaks and epidemics.

**Methods:**

This is a descriptive study carried out using secondary data from Guardians of Health available in a public digital repository. Based on syndromic signals, the information subsidy for decision making by policy makers and health managers becomes more dynamic and assertive. This type of information source can be used as an early route to understand the epidemiological scenario.

**Results:**

The main result of this research was demonstrating the use of the participatory surveillance platform as an additional source of information for the epidemiological surveillance performed in Brazil during a mass gathering. The platform Guardians of Health had 7848 users who generated 12,746 reports about their health status. Among these reports, the following were identified: 161 users with diarrheal syndrome, 68 users with respiratory syndrome, and 145 users with rash syndrome.

**Conclusions:**

It is hoped that epidemiological surveillance professionals, researchers, managers, and workers become aware of, and allow themselves to use, new tools that improve information management for decision making and knowledge production. This way, we may follow the path for a more intelligent, efficient, and pragmatic disease control system.

## Introduction

Participatory surveillance has been a reality in many parts of the world, improving traditional health surveillance systems and engaging the population to build epidemiological scenarios [1-8]. The use of mobile devices to improve the data collection, processing, and analysis processes for epidemiology and surveillance has contributed to great advances in public health in the aspects of innovation and digital transformation in this area [9-15]. With the evolution and ubiquity of mobile devices and their operating systems, in addition to increasing digital inclusion and internet connectivity, research and collaborative strategies have been adopted to improve the quality of information generated in health, especially in the understanding of epidemiological patterns [16-18]. Strategies for monitoring respiratory, diarrheal, and rash syndromes due to arboviruses are examples of how to drive digital disease detection platforms to address the production of strategic information for health surveillance based on crowdsourcing in the American, European, African, and Asian continents; some platforms include Flu Near You, Influenza.Net, AfyaData, Vigilant-e, Saúde na Copa, and Guardians of Health [4,19-29]. The use of crowdsource-based platforms has also been observed in foodborne disease surveillance, which has enabled the anticipation of disease outbreak detection and evaluation of policies for food safety, as is the case for the website iwaspoisoned.com [30].

Participatory surveillance systems usually work in similar ways, where the user is able to make a self-report of symptoms periodically. The periodicity varies from daily to weekly frequency. The collected data—symptoms and geolocation—are sent to cloud-computing servers, where data points are processed and analyzed. From the extracted data, epidemiologists, researchers, data scientists, and government agents analyze the information identifying the distribution of people with symptoms during a certain time frame [3]. Usually the approach adopted is syndromic (ie, the data collection is specific for symptoms that make up groups of diseases), thus calibrating the sensitivity and specificity of the systems. Some platforms, such as Saúde na Copa [22], Flu Near You [16], or FluTracking [31], use engagement strategies to ensure the user's adherence for regular participation within the system. In the first example, gamification was used; during the 2014 World Cup, users could play a soccer-themed game, where evolution within the game was conditional on users’ health reports. In the second example, nudges, such as referring a friend, showing the number of active users by region, awarding heavy users with badges, push notifications, and email reminders, were used to encourage participation.

Parallel to the advances of participatory surveillance, some health outcomes still need more intelligent and agile monitoring, such as the case of mass gatherings. Mass gatherings are situations involving large numbers of people participating in a specific cause, planned or not, related to leisure, religion, politics, sports, and other reasons [32].

Leal Neto et al [22] pointed out the use of participatory surveillance in mass gatherings for the first time during the 2014 FIFA World Cup, where a mobile app based on crowdsourcing was developed to identify health threats and was officially used by the Brazilian Ministry of Health. With this experience, and with the aim of improvement, a new platform for participatory surveillance in mass gatherings was developed and adopted, this time focusing on the 2016 Olympic Games [8]. Initiatives conducted by other countries during the 2016 Olympic Games were carried out, demonstrating the understanding of the importance of new participatory surveillance approaches using mobile devices to serve as an additional support for traditional health systems [33]. In addition, this work pointed out the relevance of this approach in finding outbreaks in a faster way [34].

This work aims to describe the participatory surveillance platform Guardians of Health, a project conducted during the 2016 Olympic and Paralympic Games in Rio de Janeiro, which is officially used by the Brazilian Ministry of Health for the monitoring of outbreaks and epidemics.

## Methods

### Overview

Skoll Global Threats Fund and Epitrack, with the support of the Brazilian Ministry of Health and the Pernambuco Research Support Foundation, developed a mobile app, a web app, and a dashboard platform to implement participatory surveillance in Brazil during the 2016 Olympic Games. The Rio de Janeiro-based project also included five other Brazilian cities that hosted events and games related to the 2016 Olympic Games: Manaus, Salvador, São Paulo, Brasilia, and Belo Horizonte. The platform was called Guardiões da Saúde (Guardians of Health in Portuguese). The study period was divided into a pre-event period (March 28-August 4, 2016) and an event and postevent period (August 5-October 26, 2016). The first period aligned with the time when the platform Guardians of Health was officially launched by the Brazilian Ministry of Health. The second period aligned with the occurrence of the 2016 Olympic and Paralympic Games and the 45 days following the Games.

The management structure of the platform worked across multiple centers: a base at the General Coordination of Surveillance and Response to Events of Public Health, Secretariat of Health Surveillance in the Ministry of Health in Brasilia; a base in Washington, DC, USA; and a base for development and support in Recife, Brazil.

When people downloaded the app, they were only considered as users if they agreed with the terms of use and privacy policy, checking a box before they started. The participants gave informed consent when they registered in the app. All participants were volunteers and the study caused no harm to any of them. The whole app was translated into seven languages: English, Spanish, Portuguese, French, Arabic, Chinese, and Russian. For the purpose of this work, we accessed the open data available at the platform’s project page on GitHub via Epitrack [35]. This project followed the Brazilian regulation regarding information access and handling, according to the Access to Information law (Law No. 12.527/2011). Since this project was performed by the Brazilian Ministry of Health in a nonacademic way, submission for ethical clearance was not required. The authors were involved in the system development and deployment, marketing campaigns, and user acquisition; however, the authors did not have access to participant identification or anything that could identify individual users.

### System Development

The Guardians of Health platform was composed of three segments: (1) iOS and Android apps, (2) a web app, and (3) a dashboard. The mobile app was developed using the native technology of the respective operating systems: for Android, Java was used; for iOS, Objective-C and Swift were adopted. Users who registered with information about sex, age, and city were motivated daily—by push notification, gamification, and marketing—to report their health condition. Within the options of the report, the user was able to state whether he or she was well (ie, without symptoms) or ill, where the following list of symptoms was shown, asking the user to pick one or more: body pain, headache, joint pain, cough, sore throat, fever, shortness of breath, nausea and/or vomiting, diarrhea, itching, rash, red eyes, and bleeding. The symptoms were strategically defined from a syndromic approach with the purpose of the identification of diarrheal, respiratory, and rash syndromes (see Table 1). For this project, rash syndrome was defined as symptoms related to arboviruses—dengue, Zika, and chikungunya—and the rash (ie, exanthema) symptom was necessary. For diarrheal syndrome, the diarrhea symptom was mandatory. For respiratory syndrome, cough and fever were mandatory symptoms.

Registrations and self-reports were completed and sent to the database. Their geographic coordinates—latitude and longitude per Universal Transverse Mercator—were then collected, favoring the geolocation of the records within the app. For devices that did not allow location tracking, we used the proxy location of the users’ Internet Protocols (IPs). The list of symptoms was accompanied by three more questions: (1) Did the user have contact with anyone with these symptoms? This served to establish possible links for eventual chain of transmission, (2) Did the user seek a health service to understand the severity of reported symptoms? and (3) Did the user travel abroad? This served to determine the possible introduction of acquired disease outside Brazil.

The choices of symptoms and syndrome definitions were based on guidelines from the Brazilian Ministry of Health, with aspects of the Information System of Notifiable Diseases’ daily routine and other prerogatives (ie, guidelines) defined by the competent technical areas of the institution. The same methods of collecting daily reports could also be done via our web app, which was developed in AngularJS, a JavaScript framework; the app could be accessed through its website [36], which is hosted on DreamHost servers. The back-end server was developed in NodeJS and the web server used Nginx; both ran on a Linux Ubuntu server. As a third-party application programming interface (API), we used Google Maps and Git as versioning systems.

In addition to the mobile and web apps for collecting data via crowdsourcing, Guardians of Health also had a data visualization dashboard that provided monitoring of metrics and results acquired by the apps. The dashboard was developed in AngularJS; the JavaScript D3 library was used for the construction of the graphics. In this segment of the platform, the k-means algorithm was used to identify the syndromic clusters, considering the distribution of the points in the territory and a predefined radius.

The purpose of the dashboard was to serve as an early-warning alert system for possible syndromic clusters; epidemiological surveillance technicians used the dashboard to monitor epidemiological patterns and possible disease outbreaks. To this end, component scripts and algorithms were developed in R, version 3.2.4 (The R Foundation), which worked on the cloud using cronjobs—a time-based job scheduler—and triggered changes in epidemiological patterns with color codes and alert signals.

From data reported by users, a time series was created by counting the number of events per day in each location for each of the signs and/or symptoms. One of the main challenges was to choose a technique that fit *count data*, possibly inflated by zeros, that presented small mean events per day with great variability, as observed in Saúde na Copa [22], which served as a test set for the methodologies to be applied. Given the characteristics of the data described above—count data inflated by zeros with overdispersion (ie, variance greater than the mean)—traditional methods were not applied. For instance, the *autoregressive integrated moving average* model requires data with normal average distribution; it does not reach convergence when dealing with quantities with high exponential coefficients, with many zeros, or with very small quantities, in addition to a larger set of observations. Model-based methods also exhibit the same nonconvergence constraints, even if using negative Poisson or binomial regression. Some methods, such as *cumulative sum* and *exponentially weighted moving average*, are more complex and the detection techniques for average changes can be challenging for the type of system to be implemented.

**Figure 1:**
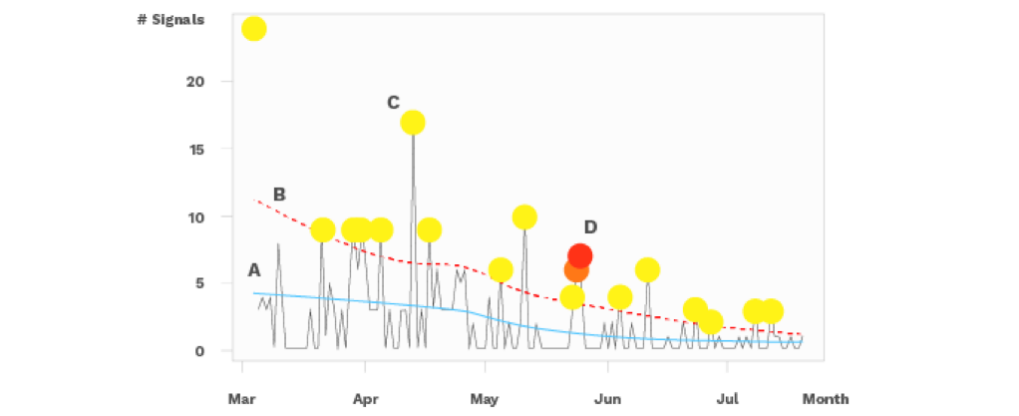
Prototype output implementation of the warning system used in the dashboard of the Guardians of Health platform. The line in blue (A) represents the locally estimated scatterplot smoothing (loess) function with a certain window; this value is known as "span" and controls the smoothness. If it is 1, the line will almost equal the mean value of the series; if it is close to zero, each point will be interpolated by the function. Thus, the function is adjusted by varying this parameter between 0 and 1. The dotted line in red (B) represents the upper range of the loess function; this amplitude was obtained by multiplying the SD by a sigma. Thus, the variability of the series decreases, which makes the interval closer to the loess function.In the event of the graduation of alerts, C indicates one of the points where the value was exceeded, but only at a moment in time which causes that point to generate a "yellow" signal. At D, the observed values exceeded the upper limit three times, the first time generating a "yellow" alert, the second time an "orange" alert, and finally a "red" alert. Then the number of cases fell below the limit and no more alarms were triggered. If this high value persisted, the alarm would have remained "red." A span of 0.75 and a sigma of 1 were used.

For these reasons, we chose an ad hoc approach by constructing a strategy to monitor signals from the Guardians of Health using the *locally estimated scatterplot smoothing* (loess) function in R. This technique uses a semiparametric local regression method, which has a smoothing parameter that defines how the function will be adjusted to the observed data. In addition to the smoothing parameter, we can also manipulate the confidence interval of the smoothed function; both parameters are easy to understand and, together with a simple rule, we have created the algorithm described below. From the series of signals, a *loess* is defined at the points passed and an associated confidence interval is established. Thus, the data arriving at the processing server can be checked against the series, and when a value passes the upper interval as defined by the parameters, a *yellow* alert is triggered. If the next point continues to exceed the upper limit, an *orange* alert is triggered. If the limit is exceeded three or more times, a *red* alert is triggered. Figure 1 shows the prototype output and simulation from the algorithm that we used.

It is important to note that because loess is a local regression method, a slow increase in cases also leads to an increase, albeit with a delay, in the upper range. This allows a very gradual increase in reported cases to be viewed as an *average process increase*, such as more people entering the system, so we have an increase in reported cases. Another important point is that as we adjust the parameters, possibly having different values for each signal or for each syndrome, we can calibrate the alert. If it is very sensitive we can reduce the number of alerts, and if the trend is rising slowly we can choose to have an alarm with a shorter latent memory.

The whole system used an API developed using the Sails framework that communicates with representational state transfer technology API (REST API). The database model used was MongoDB and the system was hosted by Amazon Web Services.

**Table 1:** Relationship between symptoms and syndromes.

Symptom	Diarrheal syndrome	Respiratory syndrome	Arbovirus (ie, rash) syndrome
Body pain	—^a^	—	Optional
Headache	—	—	Optional
Joint pain	—	—	Optional
Cough	—	Mandatory	—
Sore throat	—	Optional	—
Fever	Optional	Mandatory	Optional
Shortness of breath	—	Optional	—
Nausea and/or vomiting	Optional	—	—
Diarrhea	Mandatory	—	—
Itching	—	—	Optional
Rash	—	—	Mandatory
Red eyes	—	—	Optional
Bleeding	—	—	Optional

^a^Not applicable.

### Engagement: Acquisition, Adherence, and Permanence of Users in the Platform

One of the biggest challenges for digital platforms that require users to participate via crowdsourcing in information building is recruiting and engaging them to use the apps. There are a few examples of this recruitment and engagement, such as the Waze traffic app that has 65 million active users in more than 185 countries [37]. In the case of health, numerous strategies described by Smolinski at al [8] describe how participatory surveillance platforms seek to motivate and connect more and more with users. In the case of Guardians of Health, some strategies were developed to achieve good levels of acquisition, adherence, and engagement among users.

The distribution of the app was carried out by the Apple App Store and the Android Google Play Store. Within app store profiles, criteria that favored *app search optimization* were met, including using keywords and strategic terms that would improve the positioning of apps when users were looking for health-related terms. As users could also register on the web, elements of *search engine optimization* were implemented, with the aim of improving Google's search positioning with terms related to the health scenario. In-store ratings and comments were also monitored, where a team was responsible for responding to questions, comments, and criticisms in a timely manner, thereby by improving customer relationship management.

From the perspective of marketing as a way of acquiring users, vertical tactics of launch and media buzz were developed to target the knowledge and dissemination of the app to the regions that were associated with the 2016 Olympic Games. Press conferences, for spontaneous media generation, were given and app information was placed in Brazil's main offline and online media channels, press releases, and press kits, among others. Inbound and outbound marketing approaches were developed to target the most reach, impressions, and conversions of leads as well as users. For a marketing outbound approach, Facebook Ads, Google AdWords, YouTube ads, mobile ad networks, Google paid search, and Twitter campaigns were circulated, focusing mainly on the region of Rio de Janeiro where users were impacted by the media. For inbound marketing, blog posts were used for health issues with a high number of visits, directing readers to the distribution channels of the apps.

A gamification piece within the mobile app and the web app was developed, reinforcing the goal of user engagement in a systematic and recurring way. A game was developed into the app that consisted of a quiz with more than 300 questions about health promotion issues, disease prevention, and vector-borne diseases; this brought a health education component to the active users. The questions in the quiz were prepared by the Brazilian Ministry of Health. When answering the questions, the users were presented with information and curiosities about Olympic sports and led users through an Olympic journey, bringing the theme to the digital environment and building an imaginary mindset of the Olympic Games.

The apps also featured a health guide with information on arboviruses, travelers’ health, location of emergency room units, vaccines, useful telephone numbers, drugstore locations, basic health care recommendations, and prevention of sexually transmitted infections.

One of the limitations of crowdsourcing is the reliability of information coming from users. The validation of a possible health threat, such as the beginning of an outbreak or epidemic, is made based on groups of people reporting similar symptoms in near time and space. In the analyses performed, a *spam* classification was created, which included records with eight or more symptoms reported. It was agreed that reports with these characteristics would be removed from the analyses, as they were characterized as *spam* or *noise* within the registration database.

Another element that was considered in constructing the platform was the possibility of including secondary users nested within a primary user in Guardians of Health. In this way, a family could have one primary user and other family members added as secondary users to the account.

Data from the results of downloads, registrations, and user reports within the Guardians of Health platform were analyzed using RStudio and the Exploratory.io framework. Guardians of Health is an open source and open data project available at the platform’s project page on GitHub via Epitrack [35].

## Results

**Figure 2:**
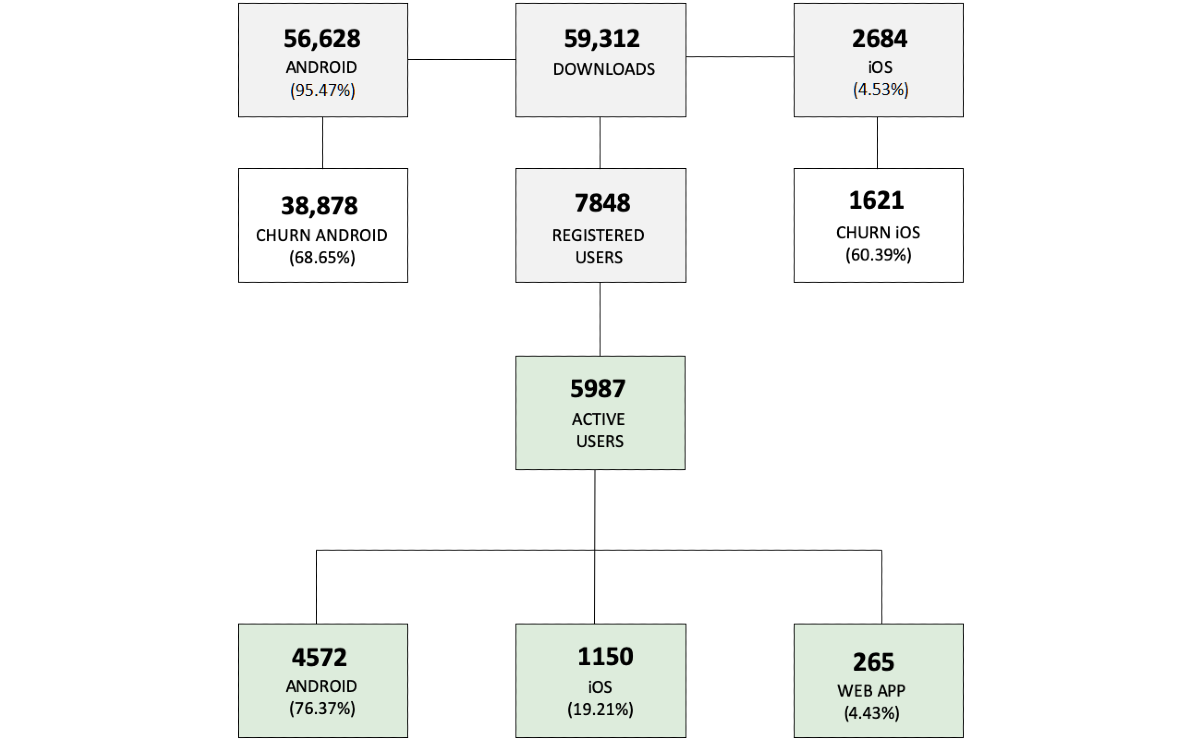
Distribution of downloads, registered users, and active users by operating system and app during the study period.

During the study period (see Figure 2), the app was downloaded 59,312 times: 95.47% (56,628/59,312) on Android devices and 4.53% (2684/59,312) on iOS devices. These downloads generated 7848 users (13.23%), where 5987 users (10.09%) sent at least one report about their health status. Of this total, 76.37% (4572/5987) were users with Android devices and 19.21% (1150/5987) were users with iOS devices. Only 265 users of the platform out of 5987 (4.43%) came via the web app. At the end of the 2016 Olympic Games period, we saw that Android device users’ churn (ie, loss of users) was 68.66% (38,878/56,628) and iOS device users’ churn was 60.39% (1621/2684).

This universe of users generated a total of 12,743 reports; after the classification and filtering of spam, this resulted in 71.79% (9148/12,743) of valid reports. A total of 80.92% (7403/9148) of reports had no symptom status and 19.08% (1745/9148) of reports presented at least one symptom in the period studied.

Regarding the users’ demographic profiles, 60.13% (5501/9148) were male and 39.89% (3649/9148) were female. The users’ ages ranged from 8 to 97 years, with a median of 39 years and a mean of 40.39 years (SD 14.06). Based on gender and ethnic policies of the Brazilian Ministry of Health, the app included a question about the race or color of the user: 30.31% (2773/9148) declared themselves as *white*, 28.54% (2611/9148) selected *black*, 21.28% (1947/9148) selected *yellow*, 18.10% (1656/9148) selected *brown*, and 1.78% (163/9148) declared themselves as *Indigenous*. Most of the reports (4601/9148, 50.30%) came from the city of Rio de Janeiro. The city of São Paulo had the second-highest number of reports (937/9148, 10.24%). Less than 5% of the reports came from other cities that had Olympic Games events, for all cities combined. Even with the platform’s promotion focused on Rio de Janeiro and other host cities, 18.89% (1728/9148) of the reports came from other cities in Brazil and, in some cases, from other countries. The average number of reports per user was 1.54 (SD 3.18, IQR 0.0) and 97.72% (8939/9148) of reports were made by the main user of the account. The average number of reports per day was 45.06 (SD 54.52).

**Figure 3:**
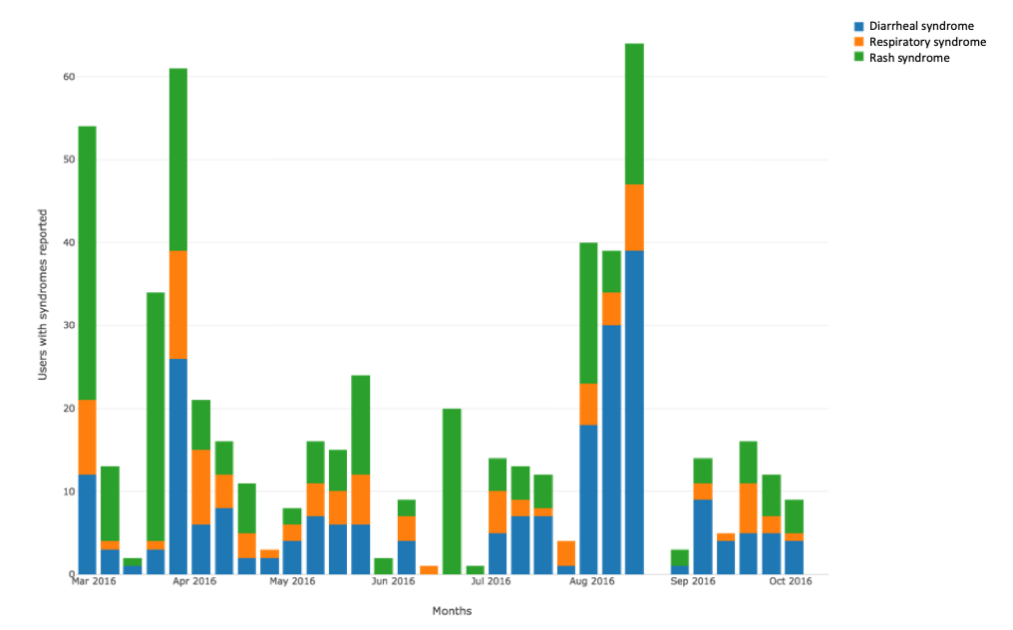
Temporal distribution for syndrome cases during the period of study.

Regarding the Guardians of Health syndromic profile, 1.76% (161/9148) of the reports were classified as diarrheal syndrome, 1.59% (145/9148) were classified as rash syndrome by arboviruses, and 0.74% (68/9148) were classified as respiratory syndrome. The frequencies of the most-reported symptoms are reported in Table 2 and the syndrome distribution is demonstrated in Figure 3.

**Figure 4:**
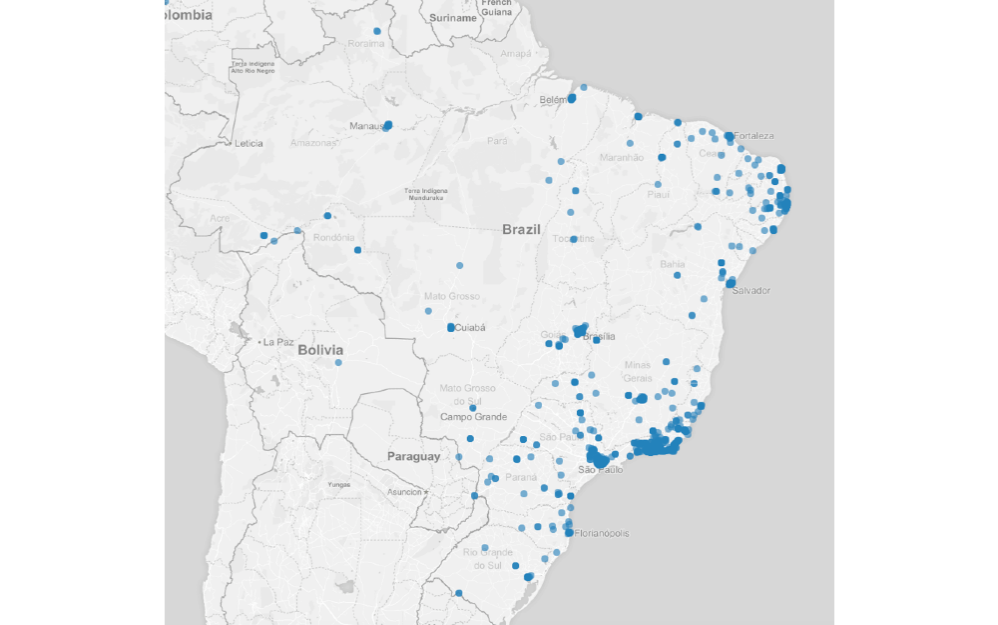
Spatial distribution of reports from Guardians of Health users with symptoms.

**Figure 5:**
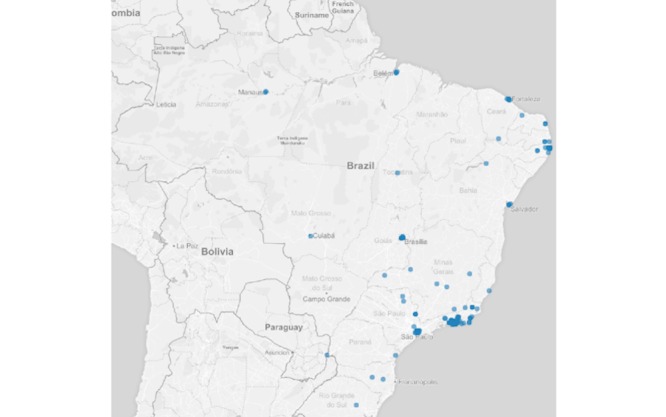
Spatial distribution of reports from users with diarrheal syndrome during the 2016 Olympic Games.

**Figure 6:**
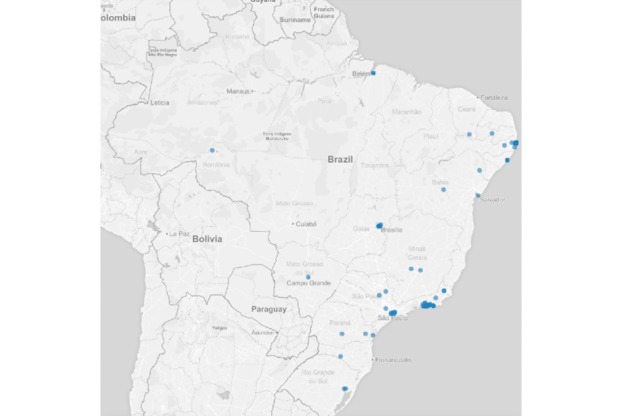
Spatial distribution of reports from users with respiratory syndrome during the 2016 Olympic Games.

**Figure 7:**
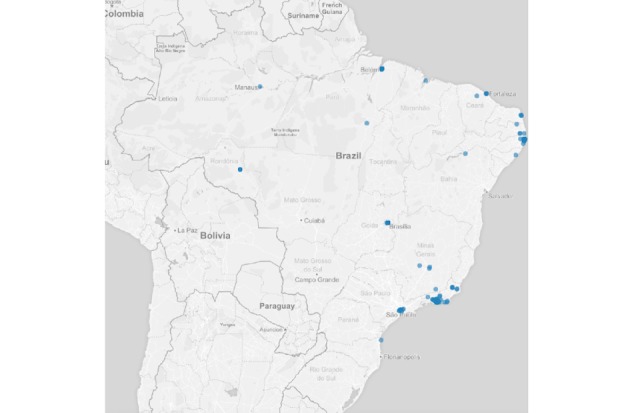
Spatial distribution of reports from users with rash syndrome during the 2016 Olympic Games.

Regarding the auxiliary questions, 1.96% (179/9148) of the reports were from users who had contact with someone with any of the symptoms described in the list. Regarding those who reported having sought health services, 2.87% (263/9148) were observed by a heath care professional. In addition, 3.05% (279/9148) of the reports were made by users who had been out of the country during the previous 2 weeks at the time of the competition. Figure 4 shows the spatial distribution of users who were feeling ill, with some symptoms. Figures 5-7 show the spatial distribution of reports that were compatible with diarrheal, respiratory, and rash syndromes, respectively.

Throughout the study period, whether during the pre-event period or during the 2016 Olympic Games, despite the evidence of reports compatible with the syndromes described above, there was no concentration of these reports in the same space and time; this excluded the possibility of the beginning of outbreaks, according to the information collected by the Guardians of Health. We have tested this using k-means and the Hartigan-Wong algorithm.

Regarding the results obtained via the engagement strategies, marketing campaigns were made through the study’s YouTube channel to deliver video campaigns about the app, which were viewed 253,061 times. Regarding Facebook, content-placement strategies on the study’s own fan page were adopted, leveraging 439 followers. However, Facebook also posted on partner pages, such *Razões para Acreditar*, which has 692,297 followers, with Guardians of Health content being disseminated through these vehicles. Regarding scores on app stores, the Guardians of Health app averaged 4.1 out of 5.0 in the Google Play Store and 3.0 out of 5.0 in the Apple App Store.

**Table 2:** List of symptoms reported via Guardians of Health during the Rio 2016 Olympic Games period.

Symptom	Number of reports (N=1746 users with at least one symptom), n (%)
Body pain	607 (34.77)
Headache	593 (33.96)
Joint pain	487 (27.89)
Cough	419 (24.00)
Sore throat	277 (15.86)
Fever	269 (15.41)
Shortness of breath	218 (12.76)
Nausea	204 (11.68)
Diarrhea	161 (9.22)
Itching	145 (8.30)
Rash	145 (8.30)
Red eyes	132 (7.56)
Bleeding	57 (3.26)

## Discussion

Participatory surveillance continues to be an alternative for health services innovation in the digital age, spreading to various parts of the world and increasingly gaining strength as an additional platform to traditional epidemiological surveillance systems [3,8,22]. The use of this platform on a national scale in Brazil during the 2014 FIFA World Cup and the 2016 Olympic Games shows that the government is willing to take a new position regarding disease surveillance; combining tools can bring modernization and substantial improvements to the sensitivity and specificity of the production and consumer structures of strategic information in health surveillance.

Experiences using mobile devices take advantage of the population's access to smartphones; this facilitates the spreading of projects that are based on real-time data collection with remote transmission of information and dashboards to support decision making [10,38-41]. In the case of Guardians of Health, for the period of operation, the high number of downloads was not effectively converted into the creation of users, where 13.23% of all who downloaded the app became registered users. This behavior can be explained with some hypotheses, such as the innovative potential of the tool, invoking the criterion of *deceptive* growth [11], that is, the incredulity of the population in something more modern than the traditional systems. The tone and sentiment observed in the comments at the virtual stores and on Facebook point to another hypothesis linked to political instability at that time in Brazil.

There was a majority of users who used the Android operating system compared to the iOS operating system. The direct explanation is the lower cost of smartphones with the Android operating system in Brazil, in keeping with the financial reality of the population. Gotz et al [39] questioned whether there was a difference in personality between the users of both systems, but no relevant results were found to support this. Therefore, it can be suggested that the economic factor involved in the acquisition cost of these devices is what generated the greater preference for Android, at least in Brazil. Data show that Brazil currently has 77.35 million smartphone users, where 92.6% are Android users [42]. The low number of registrations and use via the web app implies that internet apps are more restricted to mobile devices, as compared to desktops or access by browsers. The churn achieved by the apps was within an expected value for apps in the health field, which is 75% [43].

Using a rule to identify spam posts [22], 28.21% of reports that were not true or had a high chance of being fallacious were eliminated. Even with this withdrawal, obtaining 9148 reports generated a reasonable amount of data for analysis. There were differences between the demographic profiles identified in the Guardians of Health platform and the Saúde na Copa app, which is a participatory surveillance app used in Brazil during the 2014 World Cup. Guardians of Health showed an overlap of approximately 20 percentage points in relation to male users. There was a difference in the age of users as well, where Saúde na Copa had a smaller age range (12-77 years) than did Guardians of Health (8-97 years). One of the elements that can explain this difference is that Guardians of Health had the functionality of adding secondary users to a primary account. That is, an adult at the median age observed (39 years) could have children or the elderly as secondary users within their account. This may also explain the difference in means achieved between the World Cup experience and the Olympic Games [22].

The population profile according to race or color was a variable used for the first time in any participatory surveillance platforms, worldwide; this prevented a comparison of this profile with other regions, even aiming to raise discussions about access to technology according to race or color. However, due to the fact that Brazil has a well-known population with a majority of Europeans and Africans, while preserving Indigenous characteristics in several regions of the country, the figures presented reflect a reality of race diversity at the national level, even those who declare themselves to have represented only 1.79% of the reports.

Most of the Olympic and Paralympic competitions were held in Rio de Janeiro, explaining that most of the reports came from this locality. One situation that has been repeated in relation to the Saúde na Copa app [22] is the demonstration of the potential for scale and dissemination of a participatory surveillance platform for mobile devices. In the Guardians of Health platform, 30.83% of the reports came from Brazilian cities or from foreign territories—the minority—that were not the headquarters of any Olympic event.

The results on the captured syndromic profiles (ie, reports that were compatible with the rules of a priori defined syndromes) were below those observed in previous experiences during mass events using participatory surveillance. However, they still show great potential for use because of their sensitivity in locally identifying concentrations of reports with similar characteristics of symptoms. Fortunately, there were no outbreaks detected by the app and this was corroborated with official information from the Brazilian Ministry of Health, assuming validation of the tool's potential in the timely detection of health threats.

Unlike the Saúde na Copa app, which had peaks of participation during matches of the Brazilian soccer team, the Olympics had a diversity of sports activities; it was possible that the peaks in the number of participating users and completion of reports were related to the days when the marketing campaigns for acquisition and adherence of users took place.

Mass gatherings continue to be sensitive situations in health management, due to the pressure caused within local systems; a sudden increase in demand of resources with a structure unable to keep up with the scale of supply; as well as the epidemiological risks of introducing eradicated, new, or nonendemic diseases as well as controlled diseases into the national context. Initiatives like Guardians of Health help to track risk factors for epidemics and diseases outbreaks. Even when these risks occur during mass gatherings, such platforms are able to minimize these health threats.

Engaging users on platforms such as this remains one of the puzzling and challenging issues in terms of participatory surveillance. Building on the importance of understanding health information around them has been a quest for various groups working with participatory surveillance around the world; despite experiences with interesting success (ie, FluTracking [31]), a replicable path has not been found, making this a negative aspect in applying a participatory approach [44-46].

Another sensitive point that comes up on the list of challenges is the role of government and its agents as users and system managers. The importance of a prospective mindset is vital to foster abundant, innovation-oriented thinking, in order to improve and sustain these initiatives. It would be ideal for each experience, whether during the World Cup or the Olympic Games, to have, at minimum, a permanent structure of management, development, support, and dissemination that would favor growth more and more. However, these skewed interests and lack of agile capacity, which are needed to structure the sectors that managed to maintain projects like this, are also characterized as enigmatic challenges; a solution needs to be found to articulate and implement these experiences as a permanent part of innovative health policy.

In this scenario of the information capture flow for epidemiological surveillance, sick individuals are only counted by the health system upon entry to the system, which is then notified when necessary. However, the time between illness and demand for health care, assuming the health facility would report all relevant cases, demonstrates the fragility of traditional systems regarding the timely identification of diseases that can impact public health in the form of outbreaks and epidemics. One way to fill this gap between illness from a disease with outbreak potential and the record of it is the use of technologies and strategies, such as participatory surveillance; these empower citizens by making them an active part of joint information building, which contributes to the epidemiological setting of their community or region and rescues the precepts of social control widely debated in the Sistema Único de Saúde, the Brazilian National Health System.

Reaffirming the debate [12,13], the explicit evidence that disruptive innovations in public health are far more present in our national context than we imagine, because of their potential exponential growth, urges research and services to consider this new movement in their collective health practices. In this way, Sistema Único de Saúde can not only follow but can become an effective actor in this rapid transformation in the use of information to improve the quality of life for all citizens.

Many other participatory surveillance strategies keep appearing across the globe, using the same mindset where people are the primary data source, contributing to build epidemiological settings with crowdsourcing [47-51]. On the other hand, nontraditional approaches for health communication should be considered to work alongside traditional methods in order to increase the range of digital health, for instance, the use of YouTube to spread health education content [52,53].

Digital transformation is a fact; it is no longer a futuristic element, but has become today's reality. The world is changing fast and recognizing this transformation is essential to face today’s challenges. The struggle against those who continue to ignore this change encumbers the process of transformation, holding all those working in the context of public health hostage to obsolescence. It is hoped that professionals, new and old, as well as researchers, managers, and workers involved in epidemiological surveillance become aware and allow themselves to implement new tools that improve information management for decision making and knowledge production.

## Abbreviations

API: application programming interface
loess: locally estimated scatterplot smoothing
REST API: representational state transfer technology application programming interface
